# Network analysis of potential risk genes for psoriasis

**DOI:** 10.1186/s41065-021-00186-w

**Published:** 2021-06-16

**Authors:** Huilin Wang, Wenjun Chen, Jin He, Wenjuan Xu, Jiangwei Liu

**Affiliations:** Department of Dermatology, General Hospital of Xinjiang Military Command, No. 359 Youhao North Road, Saybak District, Urumqi, 830001 Xinjiang China

**Keywords:** Psoriasis, Differentially expressed genes, Differentially methylated positions, Pathogenesis

## Abstract

**Background:**

Psoriasis is a complex chronic inflammatory skin disease. The aim of this study was to analyze potential risk genes and molecular mechanisms associated with psoriasis.

**Methods:**

GSE54456, GSE114286, and GSE121212 were collected from gene expression omnibus (GEO) database. Differentially expressed genes (DEGs) between psoriasis and controls were screened respectively in three datasets and common DEGs were obtained. The biological role of common DEGs were identified by enrichment analysis. Hub genes were identified using protein–protein interaction (PPI) networks and their risk for psoriasis was evaluated through logistic regression analysis. Moreover, differentially methylated positions (DMPs) between psoriasis and controls were obtained in the GSE115797 dataset. Methylation markers were identified after comparison with the common genes.

**Results:**

A total of 118 common DEGs were identified, which were mainly involved in keratinocyte differentiation and IL-17 signaling pathway. Through PPI network, we identified top 10 degrees as hub genes. Among them, high expression of CXCL9 and SPRR1B may be risk factors for psoriasis. In addition, we selected 10 methylation-modified genes with the higher area under receiver operating characteristic curve (AUC) value as methylation markers. Nomogram showed that TGM6 and S100A9 may be associated with an increased risk of psoriasis.

**Conclusion:**

This suggests that immune and inflammatory responses are active in keratinocytes of psoriatic skin. CXCL9, SPRR1B, TGM6 and S100A9 may be potential targets for the diagnosis and treatment of psoriasis.

## Introduction

Psoriasis is an immune-mediated inflammatory skin disease with important physiological and psychosocial consequences [1]. Psoriasis has complex characteristics, affecting about 2% of the general population, and the prevalence of psoriasis varies from country to country [2, 3]. There are several clinical variants of this disease, namely plaque psoriasis, erythrodermic psoriasis and pustular psoriasis, among which plaque psoriasis accounts for more than 90% [4]. Clinically, red patches of silver-white multilayered scales are characteristic of psoriasis, and the patient's epidermis is thickened, clearly demarcating from adjacent non-banded skin [5]. The disease affects more than 10% of the body surface area and usually requires systemic drug therapy.

Inflammatory infiltration consisting of dermal dendritic cells, macrophages, T cells, and neutrophils is present in psoriatic plaques [6]. Help T cells have long been recognized as an important pathogenic factor in psoriasis. Further evidence suggests that there is a strong T cell component to maintain psoriasis through Th1, Th17, and Th22 cells and their derived cytokines [7]. The complex interaction of pro-inflammatory cytokines, chemokines, growth factors and chemical mediators initiated by Th17 cells may be the key factors to induce keratinocyte proliferation, angiogenesis and neutrophil influx, ultimately leading to keratinocyte over-proliferation and the characteristics of psoriatic plaques [8]. Recently, mast cells have been shown to be the major producers of interleukin-22 in psoriasis and atomic dermatitis [9]. Among various molecules associated with psoriasis, tumor necrosis factor (TNF)-α, IL-23, IL-17 and IL-22 are important regulators of psoriasis [10]. Emerging evidence suggests that different phenotypes have different immunogenetic characteristics, which may affect treatment options [11].

Epidermal gene modification is considered as an essential factor in the pathogenesis of psoriasis [12]. Epigenetic changes also play an important role in the differentiation of CD4( +) T lymphocyte subsets and in the pathogenesis of psoriasis [13]. Among all epigenetic mechanisms, DNA methylation is one of the important factors in keratinocyte differentiation [14, 15]. Common drugs used to treat psoriasis, such as methotrexate, have been reported to interfere with the methyl transfer function of folic acid, thereby restoring normal methylation status [16]. However, researchers still need to make greater efforts to elucidate how abnormal epigenetic modifications affect the pathogenesis of psoriasis. In addition, some environmental factors, including modifiable variables such as physical trauma, drug reversal, psychological stress, obesity, are associated with the development and deterioration of psoriasis [17].

In this study, psoriasis-related sequencing data in public databases were used to explore the molecular mechanisms and potential risk factors of the disease.

## Materials and methods

### Data collection

We collected psoriasis-related datasets from the gene expression omnibus (GEO) database. GSE54456 included gene expression profile from skin samples of 92 psoriatic and 82 normal punch biopsies. The genes annotated in RefSeq was used to quantify gene expression levels. The gene expression was normalized to the number of reads per kilobase per million mapped reads (RPKM). GSE114286 included gene expression profile from 9 normal skins from healthy volunteers and 18 lesional skins from patients with psoriasis. The gene expression was normalized through RPKM. GSE121212 included gene expression profile of skin tissues obtained from a carefully matched and tightly defined cohort of 28 psoriasis patients, and 38 healthy controls. Paired reads were mapped to the human reference genome (b37), number of reads for each gene was counted using HTSeq.

### Identification of differentially expressed genes

The differentially expressed genes (DEGs) between psoriasis and normal were obtained through limma R software package. The log2 fold change (FC) ≥ 2 and *P*-values < 0.05 were considered as a statistically significant difference.

### Enrichment analysis

The enrichment analysis was performed using clusterProfiler R software package for the DEGs, including gene ontology (GO) functional analysis and Kyoto Encyclopedia of Genes and Genomes (KEGG) pathway analysis. The cellular component (CC), biological process (BP) and molecular function (MF) terms belonged to GO function. *P*-value of < 0.05 was considered statistically significant.

### Construction of protein–protein interaction (PPI) network

The DEGs were put into the online tool STRING (https://string-db.org) to construct the PPI network. A combined score of ≥ 0.5 was considered significant. The hub genes were chosen based on a higher number of associations with other genes (degree) in the PPI network.

### DNA methylation analysis

GSE115797 included DNA methylation profile of 24 psoriatic disease skin tissue and adjacent normal skin samples. The signal intensities along with the detection *P*-values were calculated for each CpG probes after BMIQ normalization. Normal skinized average beta values were calculated using ChAMP R software. Differentially methylated positions (DMPs) between psoriasis and normal were achieved by limma R software package. Set screening threshold with false discovery rate (FDR) < 0.05.

### Statistical analyses

Statistical analyses were carried out with SPSS Statistics 21.0. The differences between two groups were compared by Student *t* test. LRM function in RMS R software package was used for logistic regression analysis.

## Results

### Differentially expressed genes in psoriasis

**Fig. 1:**
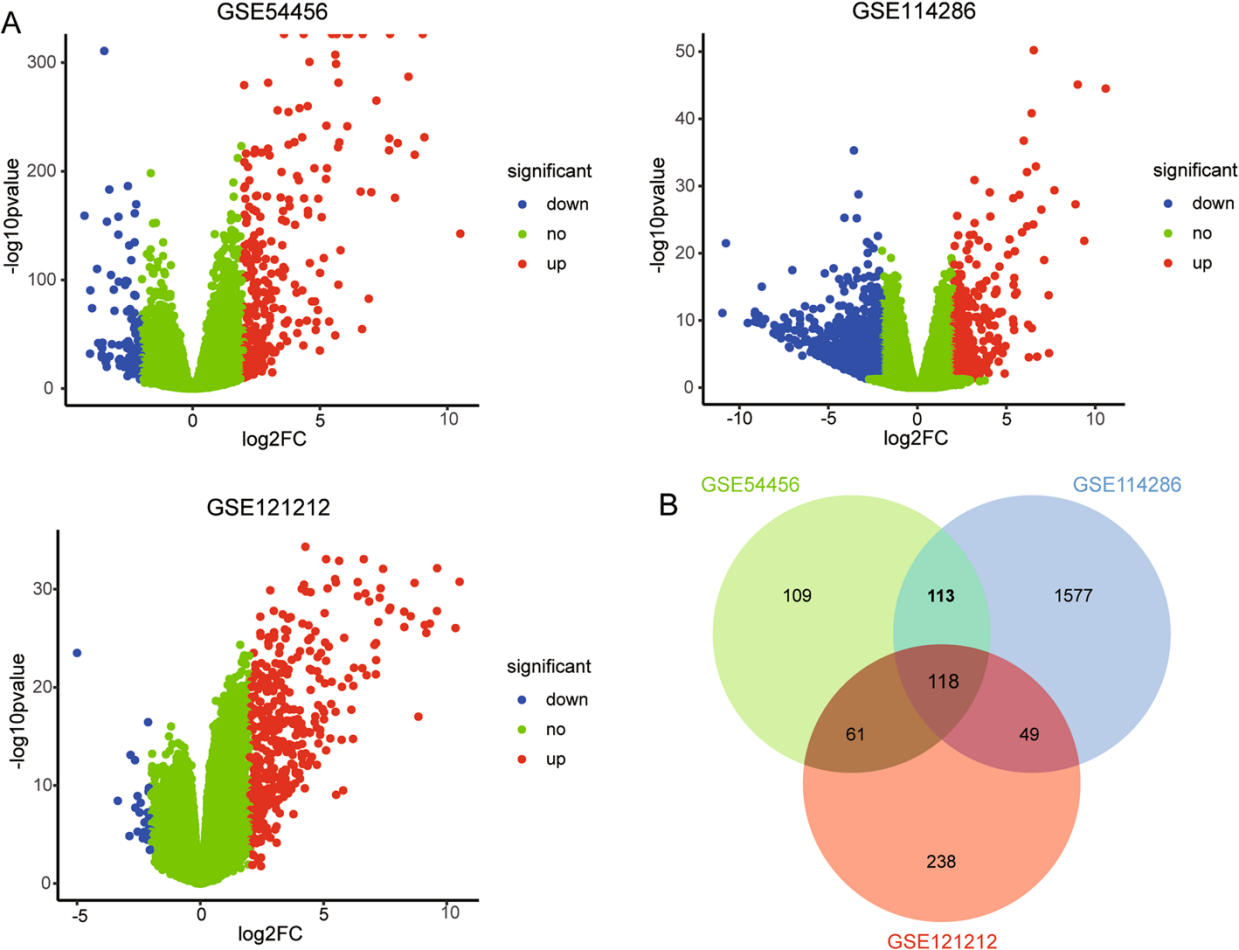
The differentially expressed genes between psoriasis and control. **A** Volcano maps of differentially expressed genes in three datasets. **B** The common genes in three groups of differentially expressed genes

By comparing the differences between psoriasis and healthy controls, we obtained differentially expressed genes (DEGs). A total of 401, 1857, and 466 DEGs were obtained in GSE54456, GSE114286 and GSE121212, respectively (Fig. 1A). Among these DEGs, we found 118 genes simultaneously present in three datasets and then defined them as common DEGs (Fig. 1B).

### Biological functions of differentially expressed genes

**Fig. 2:**
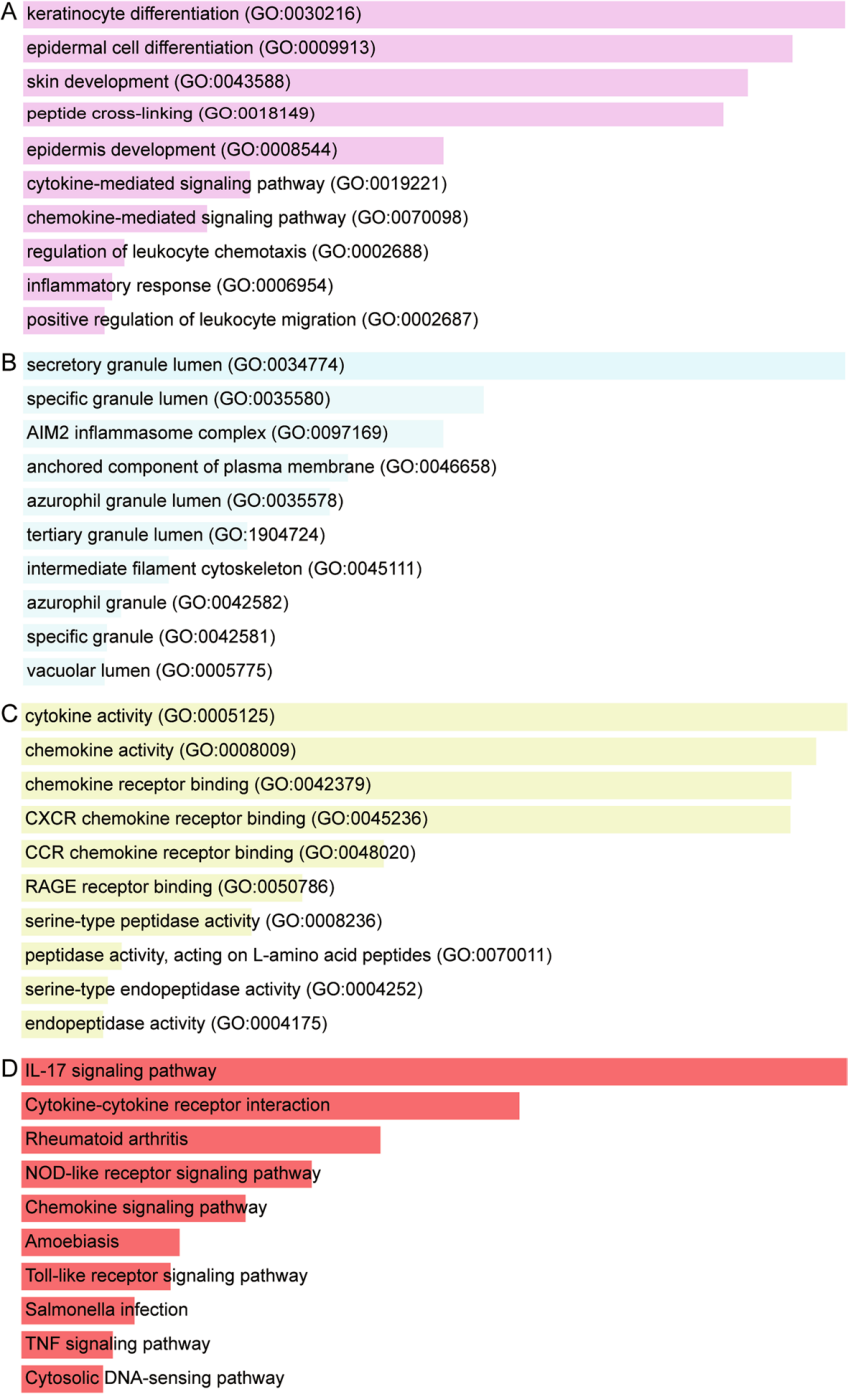
Enrichment results of common differentially expressed genes. **A** The major biological processes of common DEGs enriched. **B** The major cellular component of common DEGs enriched. **C** The major molecular function of common DEGs enriched. **D** The major KEGG signaling pathway involved by common DEGs

In the enrichment analysis results, 195 biological processes (BP), 14 cellular components (CC), and 38 molecular functions (MF) were enriched in 118 common DEGs (Fig. 2A, B, C). It mainly included keratinocyte differentiation, epidermal cell differentiation, regulation of leukocyte chemotaxis and inflammatory response. In addition, 22 KEGG signaling pathways were also enriched (Fig. 2D). Such as “IL-17 signaling pathway”, “NOD-like receptor signaling pathway”, “Toll-like receptor signaling pathway”, and “TNF signaling pathway”.

### PPI network of differentially expressed genes

**Fig. 3:**
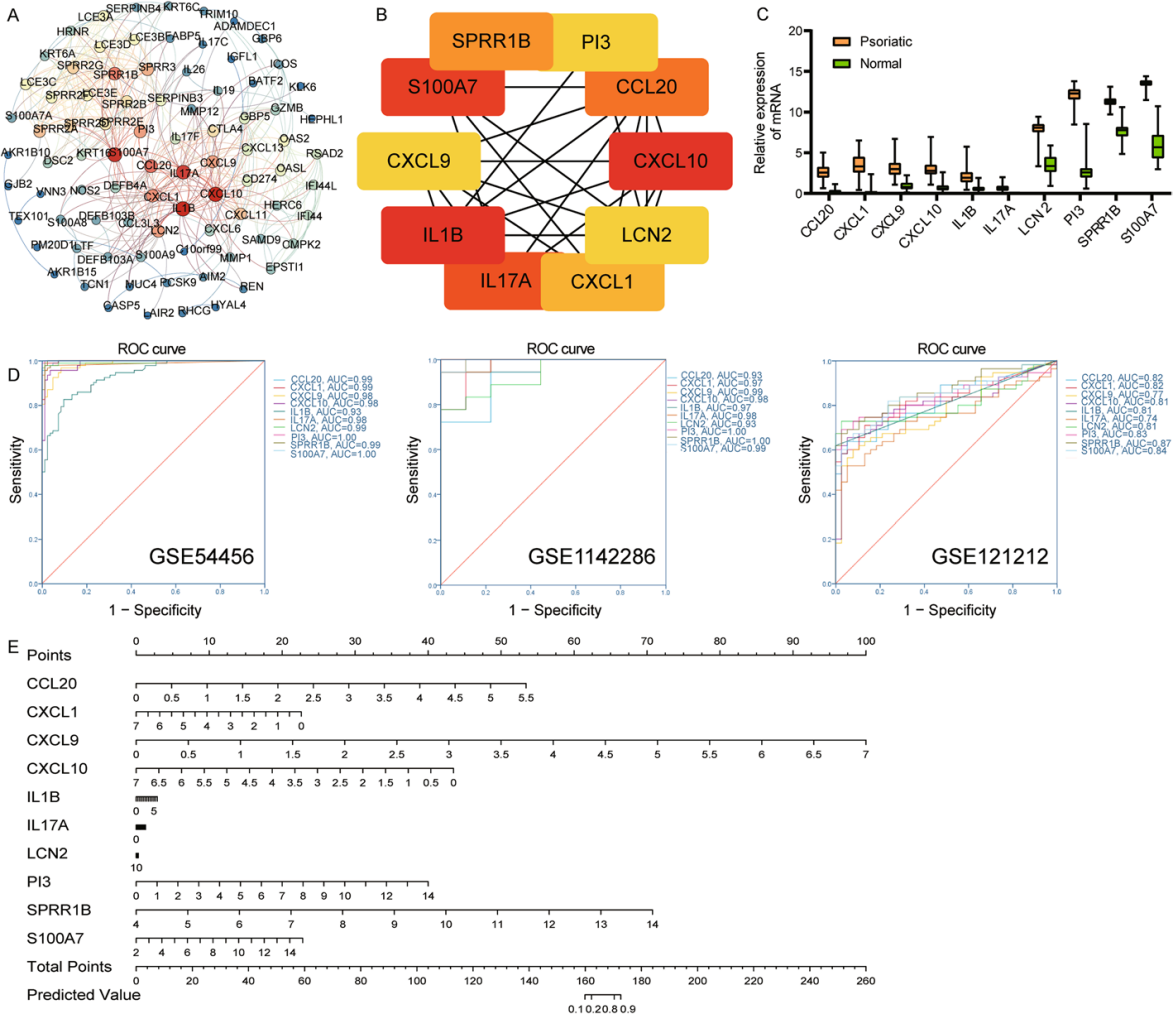
Identification of hub genes. **A** PPI network of common differentially expressed genes. **B** The top 10 10 genes with the highest degree in PPI network. **C** The differential expression of hub genes between psoriasis and controls in GSE114286. **D** ROC curve of hub genes. **E** A nomogram of hub genes predicting psoriasis risk

After screening using a combined score, a total of 86 DEGs entered to PPI network (Fig. 3A). Among them, we identified the top 10 genes with the highest degree as hub genes (Fig. 3B). Including IL1B, CXCL10, S100A7, IL17A, CCL20, SPRR1B, CXCL1, PI3, LCN2 and CXCL9. Surprisingly, they were up-regulated in all three datasets (Fig. 3C). The AUC values of hub genes were all greater than 0.7, which may have the ability to judge psoriasis, especially SPRR1B (Fig. 3D). Importantly, we performed logistic regression analysis and presented the risk prediction of hub genes for psoriasis through a nomogram (Fig. 3E). Results suggested that the higher the expression of CXCL9 and SPRR1B, the greater the risk of psoriasis. Hub genes were also mainly enriched in the IL-17 signaling pathway.

### Identification of methylation markers

**Fig. 4:**
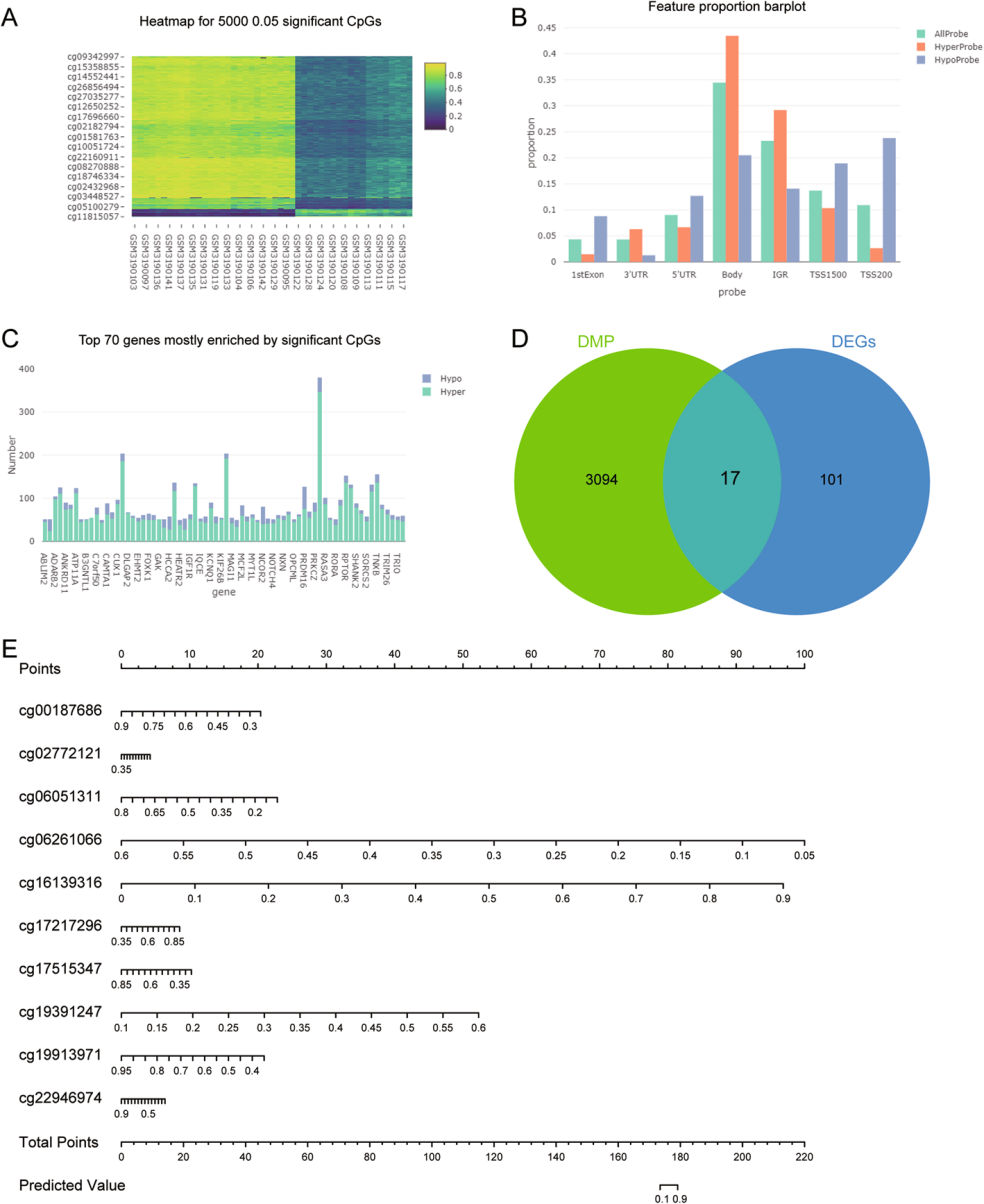
Identification of key methylation makers. **A** Heatmap of the top 5000 CpG probes with significant differences. **B** The distribution of CpG probes. **C** Hyper probe and Hypo probe of CpG in the top 70 significant differences. **D** Screening of methylation modified genes in common DEGs. **E** Nomogram of risk prediction for psoriasis through methylation markers

By analyzing the methylation modification differences between psoriasis and controls, we obtained 153,537 differential methylation probes (CpGs) for 3111 differentially methylated positions (DMPs) (Fig. 4A). These CpGs were mainly concentrated in body (Fig. 4B). Among CpGs with significant differences, HyperProbe was much more than HypoProbe (Fig. 4C). Among these DMPs, we found that 17 genes overlapped with common genes, which may be the genes regulated by methylation (Fig. 4D). Among them, CXCL1 was also the hub gene we identified. We screened the top 10 methylation markers with the higher AUC values, including cg00187686 (TCN1), cg06051311 (TRIM15), cg06261066 (TGM6), cg17217296 (AIM2), cg19913971 (TNIP3), cg22946974 (SPRR2F), cg02772121 (TRIM15), cg19391247 (TGM6), cg16139316 (S100A9), and cg17515347 (AIM2) (Table 1). The logistic regression analysis and nomogram suggested that the lower the methylation level of cg06261066 (TGM6), the higher risk for psoriasis, and the opposite was true for cg16139316 (S100A9) (Fig. 4E).

**Table 1:** The CpG Probe with top 10 AUC values

Probe	Genes	AUC	*P* value	deltBrta	FDR	logFC	*P* value
cg06051311	TRIM15	0.859	< 0.001	0.159	0.008	2.415	< 0.001
cg06261066	TGM6	0.837	< 0.001	0.192	0.004	3.215	< 0.001
cg00187686	TCN1	0.823	< 0.001	0.152	0.015	5.581	< 0.001
cg17217296	AIM2	0.823	< 0.001	0.178	0.008	3.073	< 0.001
cg19913971	TNIP3	0.819	< 0.001	0.173	0.010	5.402	< 0.001
cg22946974	SPRR2F	0.819	< 0.001	0.187	0.010	6.536	< 0.001
cg02772121	TRIM15	0.818	< 0.001	0.153	0.007	2.415	< 0.001
cg19391247	TGM6	0.818	< 0.001	0.165	0.008	3.215	< 0.001
cg16139316	S100A9	0.813	< 0.001	0.186	0.018	4.609	< 0.001
cg17515347	AIM2	0.813	< 0.001	0.174	0.009	3.073	< 0.001

## Discussion

High-throughput sequencing studies have revealed the impact of large numbers of gene expression and epigenetic modification changes on psoriasis through transcriptome and epigenetic modification studies. However, the complementarity of multiple omics data has not been fully utilized for comprehensive systematic analysis to obtain more views on disease regulation. In this study, three datasets of psoriasis were analyzed to obtain more accurate psoriasis-related genes. The molecular dysregulation mechanism and potential risk factors of psoriasis were further explored by enrichment and network analysis. Combined with methylation analysis, we obtained the methylation modified markers, which expanded the regulatory mechanism of psoriasis. In addition to previous molecular mechanism studies, our study provided a more in-depth understanding of psoriasis epidermal gene regulation.

Among the three groups of DEGs, we found 118 common genes, which may be closely related to the dysregulation mechanism of psoriasis. These DEGs were significantly involved in psoriasis-related biological functions and signaling pathways. As one of the most famous immune processes underlying the pathogenesis of psoriasis, interleukin-17 (IL-17) pathway, showed a strong enrichment effected on psoriasis-related genes and epigenetic variation [18]. In addition, there is an imbalance between the differentiation and proliferation of keratinocytes in patients with psoriasis, and IL-17A can promote the proliferation of epidermal keratinocytes [19, 20]. This promotes the development of thickened skin lesions infiltrated with a variety of inflammatory cells [21]. The inhibitory effect of anti-IL-17A on psoriasis plays an important role in the early clinical, histopathological and molecular treatment of psoriasis [22]. In general, the main function of TLRs is to mediate the inflammatory response, which has also been proved to be involved in the development of psoriasis [23]. Keratinocytes also recognize pathogens and endogenous cellular stress signals through NOD-like receptor (NLR), thereby mediating the immune response [24].

Compared with healthy controls, the hub genes we identified were up-regulated in three datasets of psoriasis. Interleukin-1β interferes with epidermal homeostasis by inducing insulin resistance, thereby participating in the development of psoriasis [25]. IL-17A activates the expression of CXCL1, CCL20 and S100A7 in keratinocytes, and activating the innate immune system [10]. The chemokines CXCL 9 and CXCL 10 released by epidermal keratinocytes have a strong chemotactic effect on the key cell monocytes, neutrophils and T cells in psoriasis [26, 27]. Consistent with our analysis, SPRR1B has also been identified as a potential biomarker of psoriasis by other studies [28, 29]. The score of psoriasis area and severity index was positively correlated with the expression of PI3 [30]. Lipocalin-2 (LCN2) is significantly higher in patients with psoriasis than in healthy controls, and may be used as a clinical indicator of psoriatic pruritus [31].

In addition, psoriasis patients have different degrees of DNA methylation compared with healthy human skin and are associated with immune activation or immunoinflammatory diseases [32]. The presence of differentially methylated positions in patients with psoriasis may be of great interest. Transglutaminase 6 (TGM6) has been reported to be associated with keratinocyte differentiation [33]. S100A9 can be an effective target for the treatment of psoriasis [34]. The level of S100A9 is related to the severity of psoriasis, which induces the production of inflammatory mediators by activating TLR 2 and 4 [35].

The limitation of this study is that we only analyzed the expression of mRNA level in limited epidermal specimens, but lacked the detection of protein expression in specimens. Secondly, we only explored the methylation modification of genes, and the specific role in the pathogenesis of psoriasis remains to be further studied. In addition, more experiments are needed to determine whether the key genes have a role in the diagnosis and treatment of psoriasis.

## Conclusion

This study identified multiple differentially expressed genes associated with psoriasis. Importantly, CXCL9 and SPRR1B, as well as methylation markers TGM6 and S100A9, have an impact on the risk of psoriasis. These genes were mainly enriched in keratinocyte differentiation and IL-17 signaling pathway. This suggests that these genes have great potential for the diagnosis and treatment of psoriasis.

## Data Availability

Data is available at NCBI GEO: GSE54456, GSE114286 and GSE121212.

## Abbreviations

AUC: Area under receiver operating characteristic curve
BP: Biological process
CC: Cellular component
DEGs: Differentially expressed genes
DMPs: Differentially methylated positions
FC: Fold change
FDR: False discovery rate
GEO: Gene expression omnibus
GO: Gene ontology
IL-17: Interleukin-17
KEGG: Kyoto Encyclopedia of Genes and Genomes
LCN2: Lipocalin-2
MF: Molecular function
NLR: NOD-like receptor
PPI: Protein–protein interaction
RPKM: Reads per kilobase per million mapped reads
TGM6: Transglutaminase 6
TNF: Tumor necrosis factor
